# Genetic Polymorphisms Affect Mouse and Human Trace Amine-Associated Receptor 1 Function

**DOI:** 10.1371/journal.pone.0152581

**Published:** 2016-03-31

**Authors:** Xiao Shi, Nicole A. R. Walter, John H. Harkness, Kim A. Neve, Robert W. Williams, Lu Lu, John K. Belknap, Amy J. Eshleman, Tamara J. Phillips, Aaron Janowsky

**Affiliations:** 1 Veterans Affairs Portland Health Care System, Portland, Oregon, United States of America; 2 The Methamphetamine Abuse Research Center, Oregon Health & Science University, Portland, Oregon, United States of America; 3 Department of Behavioral Neuroscience, Oregon Health & Science University, Portland, Oregon, United States of America; 4 Department of Genetics, Genomics and Informatics, University of Tennessee Health Science Center, Memphis, Tennessee, United States of America; 5 Jiangsu Key Laboratory of Neuroregeneration, Nantong University, Nantong, Jiangsu, China; 6 Department of Psychiatry, Oregon Health & Science University, Portland, Oregon, United States of America; University of North Texas Health Science Center, UNITED STATES

## Abstract

Methamphetamine (MA) and neurotransmitter precursors and metabolites such as tyramine, octopamine, and β-phenethylamine stimulate the G protein-coupled trace amine-associated receptor 1 (TAAR1). TAAR1 has been implicated in human conditions including obesity, schizophrenia, depression, fibromyalgia, migraine, and addiction. Additionally TAAR1 is expressed on lymphocytes and astrocytes involved in inflammation and response to infection. In brain, TAAR1 stimulation reduces synaptic dopamine availability and alters glutamatergic function. TAAR1 is also expressed at low levels in heart, and may regulate cardiovascular tone. *Taar1* knockout mice orally self-administer more MA than wild type and are insensitive to its aversive effects. DBA/2J (D2) mice express a non-synonymous single nucleotide polymorphism (SNP) in *Taar1* that does not respond to MA, and D2 mice are predisposed to high MA intake, compared to C57BL/6 (B6) mice. Here we demonstrate that endogenous agonists stimulate the recombinant B6 mouse TAAR1, but do not activate the D2 mouse receptor. Progeny of the B6XD2 (BxD) family of recombinant inbred (RI) strains have been used to characterize the genetic etiology of diseases, but contrary to expectations, BXDs derived 30–40 years ago express only the functional B6 *Taar1* allele whereas some more recently derived BXD RI strains express the D2 allele. Data indicate that the D2 mutation arose subsequent to derivation of the original RIs. Finally, we demonstrate that SNPs in human *TAAR1* alter its function, resulting in expressed, but functional, sub-functional and non-functional receptors. Our findings are important for identifying a predisposition to human diseases, as well as for developing personalized treatment options.

## Introduction

The G protein-coupled trace amine-associated receptor 1 (TAAR1) is stimulated by neurotransmitter precursors and metabolites such as β-phenethylamine (β-PEA), tyramine, synephrine and octopamine, which are present in the central nervous system at concentrations approximately 100-fold lower (0.1-10nM) than concentrations of dopamine, norepinephrine or serotonin [1], and trace amines have been reported to produce neuromodulatory effects at submicromolar concentrations [2–4]. Multiple reports describe the role of TAAR1 in physiology and behavior, including predisposition to drug abuse, as well as drug abuse-related effects. Amphetamines and drugs with disparate structures, including lysergic acid diethylamide (LSD) and amiodarone metabolites also stimulate the receptor [5–8]. *Taar1* knockout (KO) mice have an exaggerated locomotor response to MA, and the spontaneous firing rate of their dopamine neurons is increased compared to wild type mice [9, 10]. Quantitative trait locus (QTL) analysis using DNA from selectively bred MA high drinking (MAHDR) and low drinking (MALDR) mice, which were derived from a C57BL/6 (B6) x DBA2/J (D2) F2 cross identified a QTL with a large effect on chromosome 10 [11] that includes the mouse *Taar1 (mTaar1)* gene [12], suggesting that this gene plays a role in MA oral self-administration [13]. D2 mice drink more MA than B6 mice and express a non-synonymous single nucleotide polymorphism (SNP) for *mTaar1*. Furthermore, MAHDR mice are homozygous for this D2 allele, whereas MALDR mice are homozygous for the B6 allele or are heterozygous [12]. Like D2 and MAHDR mice, *Taar1* KO mice on a B6 background orally self-administer more MA than wild type mice, and importantly, the behavior is linked to a loss of sensitivity to the aversive effects of the drug in all three genotypes. In addition to inhibiting dopamine release, selective TAAR1 agonists (RO5256390, RO5263397) attenuate cocaine-induced locomotor activity, as well as activity induced by N-methyl-D-aspartate receptor agonists, suggesting that the TAAR1 influences endocrine [14] and neuropsychiatric disorders including depression, schizophrenia, and psychosis [10, 15–19].

Pharmacological evaluation of the role of TAAR1 in various behaviors is difficult because TAAR1 ligands including amphetamines and ergolines interact with other receptors and/or with neurotransmitter transporters [20]. Recently, a series of compounds that are more selective for the TAAR1, including the partial agonists RO5203648 [17, 21] and RO5263397 [18], and the full agonists RO5256390 [18] and RO5166017 [16], have been used to demonstrate that the TAAR1 is involved in drug responses that are relevant to human behaviors, possibly by altering dopaminergic and serotonergic function [16]. Qualitative drug effects on the TAAR1 are consistent across species, but quantitative effects (EC_50_ or IC_50_ values) differ, making it difficult to draw conclusions about the human response using data derived from experiments involving rodents or non-human primates [16, 22, 23]. Site-directed mutagenesis and drug effects have been helpful in modeling TAAR1 structural requirements for ligand binding and/or function [24, 25], although knowledge of which amino acid residues are required for binding of ligands and which are involved in response and regulation is not complete.

There are about 50 synonymous and 50 non-synonymous SNPs in the human *TAAR1* (*hTAAR1*), but there are no reports describing functional effects of the SNPs. A change in the function of the hTAAR1 could have important implications for detecting the predisposition to several diseases, as well as for their treatment. Here we describe SNP-induced changes in both mouse and human TAAR1 function. The mouse sequencing data disagree with predicted haplotypes based on PCR marker genotyping data and indicate that behavior-genotype correlations in RI strain mice are not valid for TAAR1. The pharmacology of the SNPs extends studies on receptor modeling, and suggests that SNPs in *hTAAR1* could provide a useful screening tool for determining the predisposition to a variety of human diseases, as well as a tool for individualizing treatments using TAAR1-specific therapies.

## Materials and Methods

### Drugs and reagents

β-PEA and tyramine were purchased from Sigma (St Louis, MO, USA). MA and LSD were generously provided by NIDA Drug Supply Program. Polyethylenimine (PEI, MW 40000) was purchased from Polysciences (Warrington, PA, USA). For experiments *in vitro*, the TAAR1 antagonist, N-(3-Ethoxy-phenyl)-4-pyrrolidin-1-yl-3-trifluoromethyl-benzamide (EPPTB) [26] was first diluted in DMSO, and subsequently diluted into cAMP assay buffer for a final DMSO concentration of 0.1%.

### Transient and stable transfection and cell culture

HEK293 cells were grown in DMEM supplemented with 10% fetal clone serum, penicillin G (100U/mL), and streptomycin sulfate (100ug/mL). Chinese hamster ovary cells (CHO-K1, ATCC) were grown in HAM/F12 medium containing 10%FCS. The plasmid DNAs containing the full-length coding region of the mouse *mTaar1* and the *hTAAR1* (including a C-terminal GFP tag) were obtained from OriGene (Rockville, MD, USA). Mutation of the *mTaar1* gene was created using the QuickChange Lightning Kit (Agilent). Mutation was verified by sequencing. Mouse constructs were transfected into HEK293 cells using Lipofectamine 2000 (Invitrogen). Stable transfectants were selected in 600μg/ml G418 and subsequently analyzed for cAMP accumulation using the EIA kit (Cayman). Similar methods were used to construct the *hTAAR1* SNPs from the reference sequence. The constructs of the *hTAAR1* that included SNPs were transiently transfected into CHO-K1 cells using PEI (1μg/μl; PEI:DNA = 1:2). cAMP accumulation assays were performed 48 hours post-transfection. Expression levels of the hTAAR1 protein, including variants, were determined by measuring GFP intensity in 10^5^ cells using a Perkin Elmer Victor X light fluorescence reader.

### cAMP accumulation assay

Cells expressing the B6- and D2-like constructs were seeded at a density of 2×10^5^ cells/well in 48-well tissue culture plates two days before an assay, with culture medium containing 10% FCS. CHO cells were plated in 24-well plates at a density of 4 X 10^5^ cells/well after transfection. One day before the assay, cells were switched to culture medium containing 10% charcoal stripped FCS and incubated overnight. Experiments were completed in assay buffer as previously described [27]. Seven concentrations of β-PEA, tyramine, LSD or MA (10^−8^ to 10^−4^) were added and cells were incubated for 60 min in the presence or absence of 10 μM EPPTB (30 min pre-incubation). cAMP accumulation was measured using a cAMP EIA kit (Cayman Chemical, Ann Arbor MI), according to the manufacturer’s instructions. All experiments were conducted with duplicate determinations.

### *Taar1* sequencing

Genomic DNA from B6, D2 and many of the older and newer BxD RI mouse strains was extracted from ear or tail tissue using QuickExtract DNA Extraction Solution (Epicentre, Madison, WI). *Taar1* DNA was PCR amplified using a HotStar Taq kit (Qiagen, Valencia, CA) with sequence specific primers surrounding the SNP-containing region (forward 5’-CACCAACTGGCTCCTTCACT-3’, reverse 5’-CGGTGCTGGTGTGAACTTTA-3’). PCR products were run on a 1.5% agarose gel, and then purified using the QIAquick gel extraction kit (Qiagen, Valencia, CA). Purified DNA was sequenced at the Oregon Health & Science University sequencing core using the forward primer to amplify the *mTaar1* gene. Sequences of PCR products were aligned and compared with *mTaar1* sequence (NM_053205.1).

### Confocal microscopy

Cells were plated on poly-L-lysine coated glass coverslips, and then fixed with 4% paraformaldehyde for 15min. Coverslips were mounted on glass slides using the Prolong Anti-fade kit (Invitrogen), and cells were imaged using a laser-scanning confocal Leica TCS SP5 microscope with 40× objective. Consecutive sections were sequentially scanned with 488 nm lasers, and sections were imaged at 0.97 μm intervals.

### Data analysis

Dose-response curves for cAMP accumulation were analyzed by nonlinear regression. Significant differences were assessed by one-way analysis of variance (ANOVA) or two-way ANOVAs of genotype by β-PEA, MA or tyramine concentration using the program GraphPAD Prism (San Diego, CA). P values < 0.05 were considered significant.

## Results

### *mTaar1* in standard inbred strains

**Fig 1:**
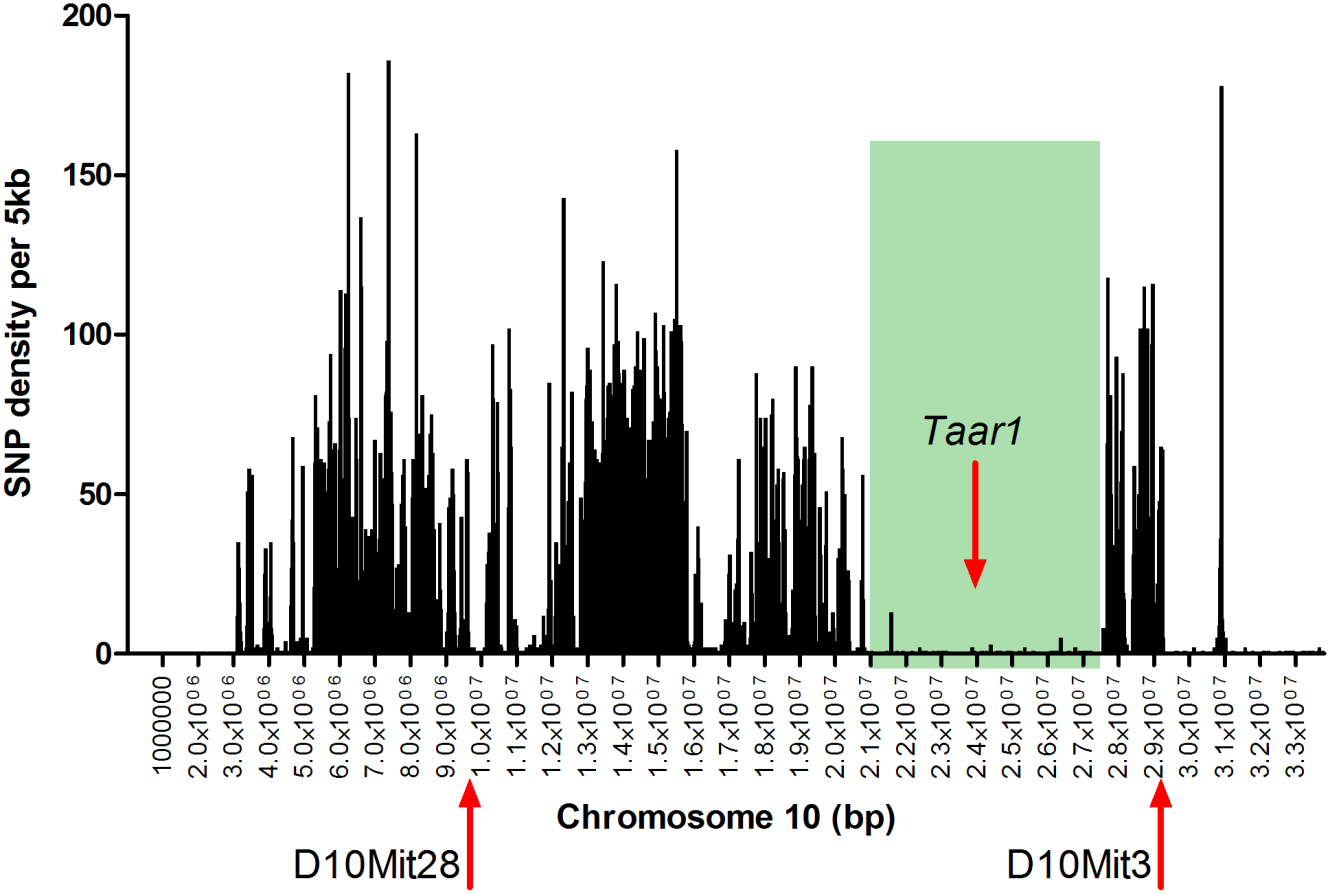
B6 vs D2 SNP density on proximal chromosome 10 (from 5 kb– 33.8 Mb). SNPs between B6 and D2 were counted in 5kb bins. D10Mit28 and D10Mit3 are the original PCR markers used in the BXD RI genotyping spanning the *mTaar1* region [29–31]. The green shading indicates the 7 Mb region in which *mTaar1* has the only D2 missense SNP.

*mTaar1* is a single exon gene with few SNPs. The 29 strains listed in Table 1 have been sequenced by the Wellcome Trust Sanger Mouse Genomes Project [28]. There are 11 missense alleles, each unique to a single strain, i.e., a rare or minor allele. Importantly, SNP, rs33645709, specific to DBA/2J (Jackson Laboratory) changes a proline to a threonine in the amino acid sequence (P77T). Also on mouse chromosome 10, is the cluster of nine trace amine receptor genes (*Taar1 –Taar9*), which spans from 23,920,387 to 24,109,564 base pairs. Within that entire genomic region, rs33645709 in *Taar1* is the only sequence variant between B6 and D2 mouse strains. There are other SNPs in *Taar2* through *Taar9* among other mouse strains, but the *Taar* cluster is in a region with few B6 versus D2 variants (Fig 1), with only 1 missense variant in over 7 Mb.

**Table 1:** Only recently derived BXD RIs have the SNP variant found in the current D2 mice. Tissue or DNA from B6, D2, and the listed BXD RIs was obtained, prepared, and sequenced as described in Methods. Only the D2 inbred mouse strain and a subset of recently derived BXD RIs express a non-synonymous SNP at position 229, resulting in a threonine to proline substitution at position 77 (P77T) in the second transmembrane domain of mTAAR1. Het: heterozygous for the B6 and D2 alleles.

Taar1 SNP: rs33645709	ACC (Thr)	CCC (Pro)
inbred lab strains	**DBA/2J** (D2, 2015)	**C57BL/6J** (B6), 129P2/OlaHsd, 129S1/SvImJ, 129S5SvEvBrd, A/J, AKR/J, BALB/cJ, BUB/BnJ, C3H/HeJ, C57BL/10J, C57BL/6NJ, C57BR/cdJ, C58/J, CBA/J, DBA/1J, FVB/NJ, I/LnJ, LP/J, NOD/ShiLtJ, NZB/B1NJ, NZO/HlLtJ, NZW/LacJ, SEA/GnJ, WSB/EiJ
wild-derived strains		SPRET/EiJ, PWK/PhJ, MOLF/EiJ, CAST/EiJ
BXD recombinant strains	BXD106, 108, 109, 115, 121 (het), 122, 123, 125, 126, 129, 138 (het), 140 (het), 145, 146, 148, 149 (het), 150 (het), 153 (het), 160, 162	BXD1, 2, 5, 6, 8, 9, 11,12,14–16, 18–25, 27–32, 43–45, 48, 48a, 51, 55, 56, 60, 63–66, 68–71, 73–75, 77, 79, 83, 84, 87, 89, 90, 98–102,105, 107, 110, 112, 116–120, 124, 127, 128, 130, 139, 142–144, 151, 152, 154, 165

### Mouse *Taar1* in BXD recombinant strains

In the interest of correlating the *mTaar1* allele with behavior, we turned to the BXD RI strains, which have been tested for thousands of phenotypes over many decades [29–31]. The PCR genotyping markers that have been used in the BXD RI strains spanning *mTaar1* are D10Mit28 at 9.413375 Mb and D10Mit3 at 29.152196 Mb (build GRCm38, Fig 1) and show a mix of B6 and D2 alleles for three earlier phases of RI strain development (derived beginning ca 1969, ca 1991, and ca 1998). For those RI strains with the same allele (B6 or D2) for both of the spanning markers, we predicted the same genotype for the *mTaar1* sequence at position 229 (Fig 1), based on B6 and D2 haplotypes (i.e., tightly linked genes on a chromosome inherited together). Surprisingly, upon sequencing *mTaar1* at position 229 in 67 of the RIs, we found only the B6 allele (Table 1). The B6 and D2 haplotypes in this region are likely maintained, meaning that the other SNPs in this region appropriately match the B6 or D2 haplotypes, indicating that the *mTaar1* mutation occurred in the D2 population at the Jackson Laboratory after the first sets of BXD RI strains were derived. To confirm that the D2 allele is a recent spontaneous mutation, as opposed to being an allele lost in the RIs due to negative selection pressure, we examined RIs BXD #105 to BXD 165 that are currently being inbred from F2 intercrosses that began ca. 2008 with new B6 and D2 breeders from the Jackson Laboratory. Table 1 indicates that some of those RIs possess the D2 SNP, which was not seen in the earlier derived RIs, confirming that the mutation is of recent origin.

### Effects of a non-synonymous SNP on agonist-induced function of mTAAR1

**Fig 2:**
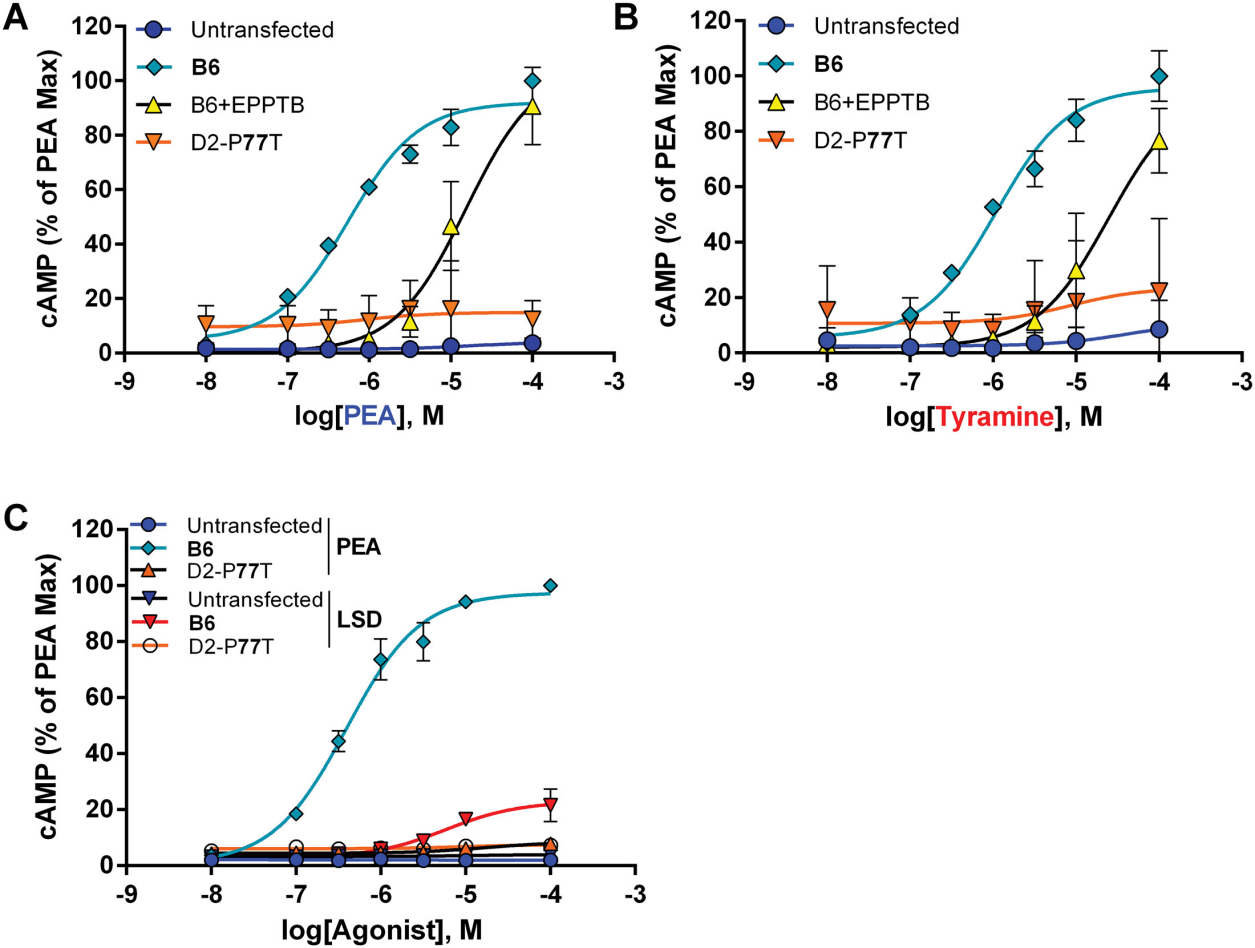
β-PEA and tyramine stimulate recombinant B6 but not D2 mTAAR1. The endogenous ligands, β-PEA (A) and tyramine (B) elicited a dose-dependent response in mammalian cells expressing recombinant B6-like, but not the D2-like (P77T) mTAAR1. Dose-response curves for the recombinant B6-like receptor were shifted to the right by EPPTB (10 μM). β-PEA EC_50_ = 0.547± 0.03 μM; Tyramine EC_50_ = 0.940 ± 0.12 μM. LSD has no effect on either receptor (C).

The effects of the P77T substitution on cAMP response to the physiological agonists β-PEA and tyramine in the mTAAR1 were examined using cells expressing the recombinant B6- and D2-like receptors. We have previously reported the effects of MA on mTAAR1-mediated cAMP production [12]. Fig 2A and 2B indicate that β-PEA and tyramine (respectively) stimulate B6 but not D2 (P77T) mTAAR1 receptor-mediated cAMP accumulation. Additionally, LSD has no effect on either receptor (Fig 2C). Drugs have a rank order of potency of β-PEA (EC_50_~547nM) > MA (EC_50_~826nM, from [12]) > tyramine (EC_50_~940nM). The TAAR1 antagonist, EPPTB, inhibited the response to agonists, shifting the β-PEA and tyramine dose-response curves to the right. Maximal response of the B6 receptor did not differ across agonists. Our previous work using confocal microscopy and western blot analyses indicates that both B6- and D2-type recombinant receptors are robustly expressed in the cytoplasm of HEK-293 cells [12]. Thus, lack of response is not due to reduced expression. Likewise, the cellular membrane is not a barrier for these agonists, which can diffuse across both artificial and synaptosomal membranes [32, 33].

### Human *TAAR1*: Effects of non-synonymous SNPs on agonist-induced function

**Fig 3:**
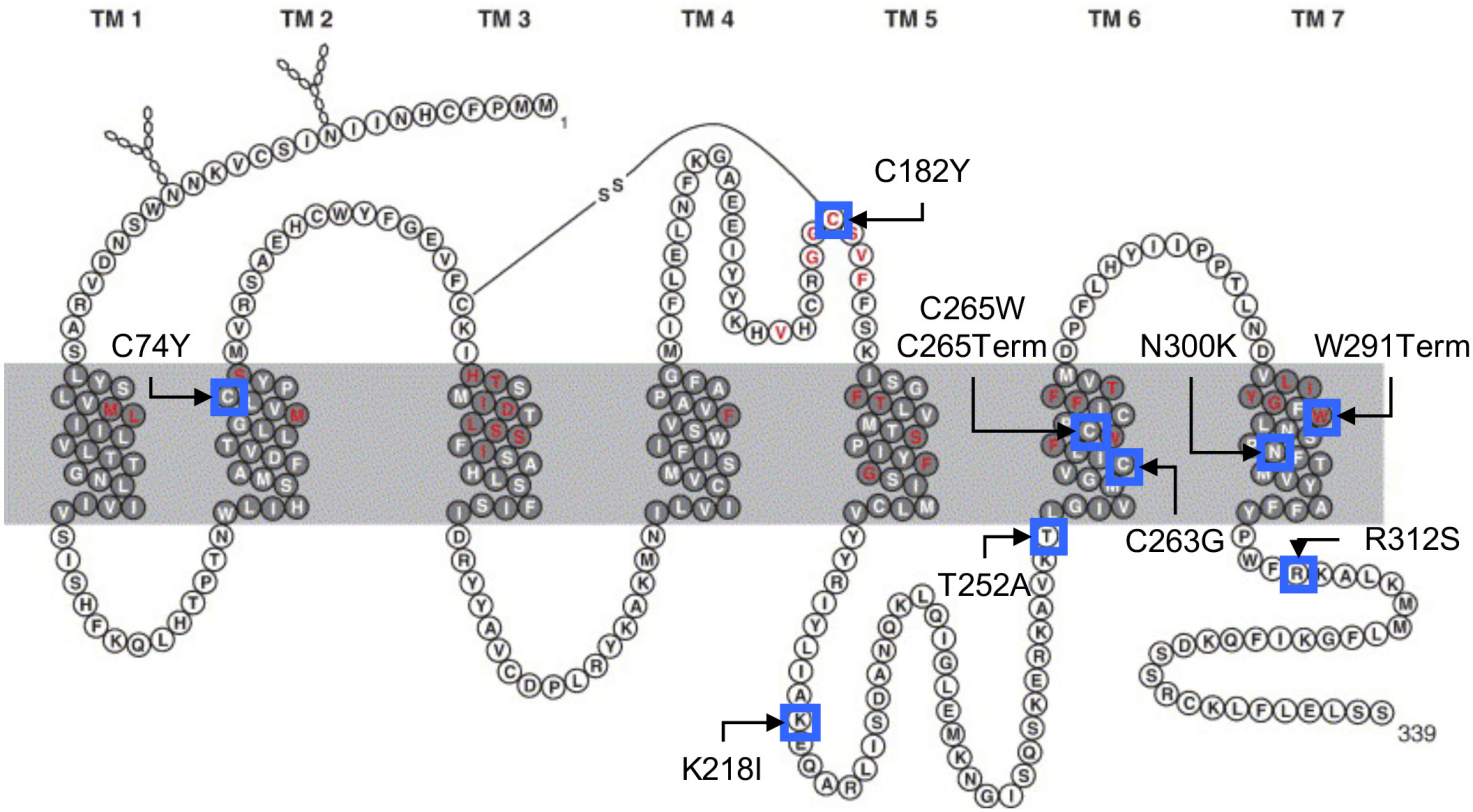
Schematic transmembrane topology of hTAAR 1. (Adapted from [35].) Amino acid residues incorporated in the transmembrane domains are shaded in gray, residues putatively involved in ligand binding are colored red. N-linked glycosylation at N 10 and N 17, as well as the disulfide bridge linking C 96 and C 182, is indicated according to the annotation in Swiss Prot entry TAR1_HUMAN. Known non-synonymous SNPs in the hTAAR1 that may produce changes in receptor function are boxed in blue (as determined from NCBI SNP Database). Indicated is the amino acid change induced by the SNP as: reference amino acid, residue number, change amino acid (Term = Termination). Further details, including the SNP nucleotide change are provided in the text.

**Fig 4:**
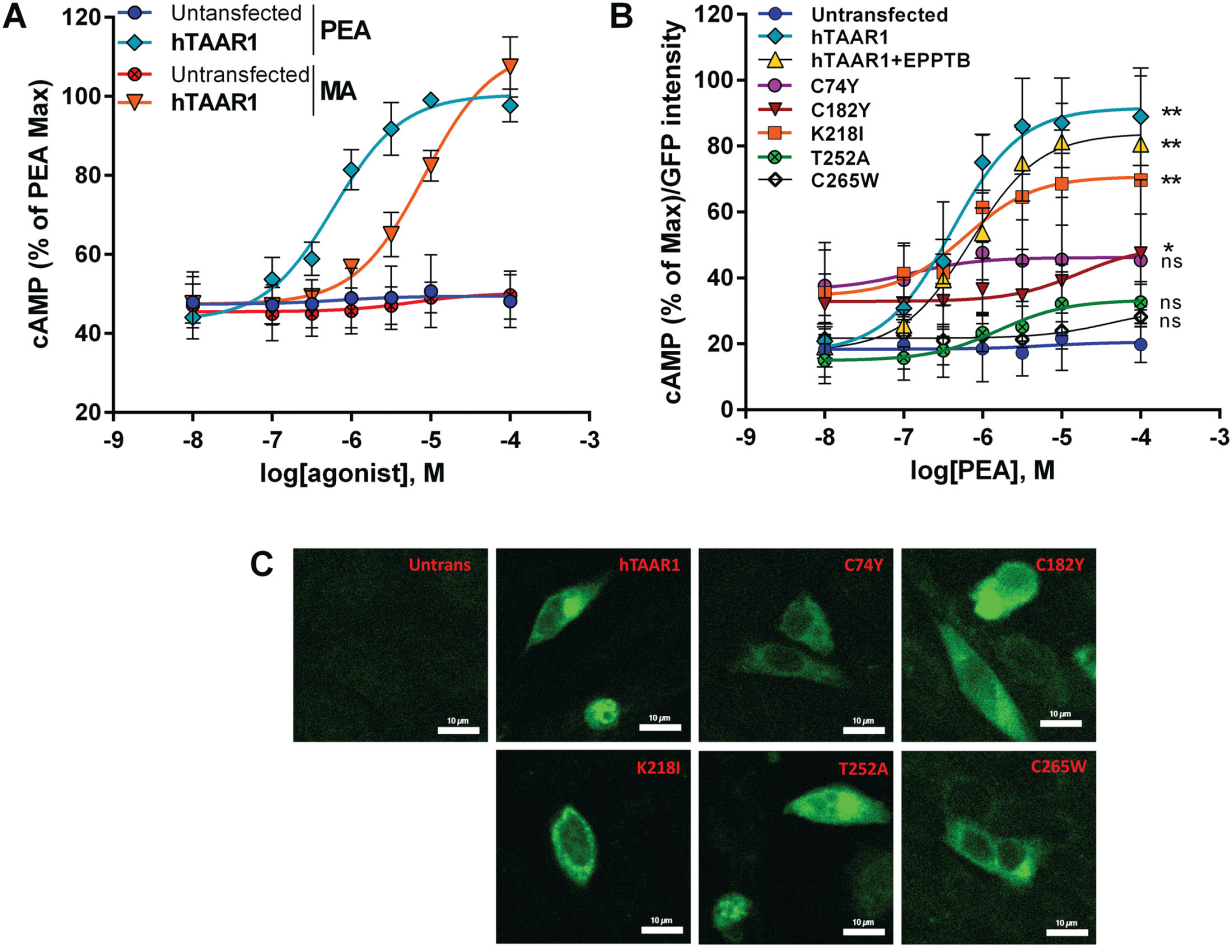
β-PEA and MA stimulate hTAAR1, and hTAAR1 SNPs reduce or eliminate receptor function. A. β-PEA and MA increased cAMP production in CHO cells expressing the hTAAR1. There was a significant dose × agonist interaction (F (18, 56) = 15.70, *p*<0.0001. *p < 0.01 for comparison between the indicated group and the untransfected control. ^+^*p* < 0.01 for comparison between PEA and MA groups. B. β-PEA dose-response curves in untransfected CHO cells and cells expressing the hTAAR1 variants. EPPTB (10 μM) caused a rightward shift in the β-PEA dose-response curve. There was a significant dose × receptor variant interaction (F (42,147) = 3.189; p<0.0001). * p<0.05 comparing C182Y and untransfected control cells; ** p<0.0001. ns = non-significant. Data shown are means ± SEMs from at least three independent experiments, each conducted with duplicate determinations. C. Confocal images of receptor expression in transfected cells.

There are close to 50 non-synonymous and 50 synonymous SNPs reported for *hTAAR1* (dbSNP database, NCBI). Eight of the SNPs were individually constructed in *TAAR1* of the reference human sequence that was GFP-tagged at the C terminus, and transfected into CHO cells. Fig 3 depicts the location of the SNPs in a schematic diagram of hTAAR1. The rationale for characterizing the selected amino acids includes the observation that transmembrane (TM) 7 mutations are in highly conserved motifs: NPXXY is one of the most highly conserved motifs in G protein-coupled receptors, and W291 is also conserved. R312 in the C terminus is part of a Phe-Arg-Lys (FRK) sequence that is conserved among catecholamine receptors including dopamine D1 and D2 receptors. The tyrosine and lysine in intracytoplasmic (IC) loop 3 are in the G protein-coupling region. The C182Y substitution destroys a highly conserved di-sulfide bridge. Thus there was a high probability for some of these SNPs to alter receptor function. hTAAR1 responded to both β-PEA (EC_50_ = 0.66 ±0.13μM) and MA (EC50 = 8.27±2.1μM) stimulation (Fig 4A). β-PEA was used to stimulate the receptors because it is more potent at hTAAR1. Fig 4B indicates that one of the chosen variants (K218I) results in a sub-functional receptor, while the variants, C74Y (a rather conservative amino acid substitution) and C265W (near the C-terminus) mediate receptor responses that are not statistically different from the response of non-transfected cells, i.e., these variants are non-functional. The nonlinear regression of the curve yielded an EC_50_ for β-PEA at C74Y and T252A of 0.12uM and 40uM, respectively. The maximum response elicited by β-PEA at C182Y was significantly different from the untransfected control, with an EC_50_ of 16uM compared with the reference hTAAR1. Additionally, the TAAR1 antagonist, EPPTB (10μM), had an inhibitory effect on cAMP accumulation in response to β-PEA by the reference hTAAR1. However, the responses of hTAAR1 to β-PEA in the presence or absence of EPPTB are not statistically different. This is consistent with reports in the literature that EPPTB is more potent at mTAAR1 compared to hTAAR1[34, 35]. As with the D2 mouse P77T SNP, confocal microscopy of GFP-tagged human receptors indicated that all were expressed in CHO cell cytoplasm (Fig 4C), so the lack of response to agonist by some variants was not due to SNP-induced loss of expression.

## Discussion

The occurrence of spontaneous mutations in the BXD RIs has been noted previously (e.g., [36, 37]). Prior to the most recent derivation of BXD RIs (ca 2008), there were three independent phases of BXD RI derivation that occurred over a period of about 30 years, and none of the sets of progeny strains express the non-functional mTAAR1 D2 variant. It is unlikely that the non-functional P77T D2 allele existed before the derivation of the original RIs, and was subsequently lost due to negative selection pressure in all three independent phases of RI derivation [36]. Rather, the mutation occurred more recently, but prior to the derivation of the more recent BXD RIs. This conclusion is supported by the latest derivation of RIs (#105–165), some of which express the non-functional variant. The derivation is still in process but began with D2 mice in ca. 2008. Therefore, the mutation arose subsequent to 1998 (after the last of the three early derivations) and before 2008. Additionally, the mutation must have occurred at The Jackson Laboratory, the source of the D2 mice used in these studies. Unfortunately, the absence of the mutation in the earlier derived RIs precludes correlation analysis with thousands of phenotypes with the *mTaar1* SNP [31]. However, the mutation segregates in the more recently derived RIs and they can be used to map other traits to *mTaar1*, and to modifiers of *mTaar1* effects. Of critical importance are the sequencing results (Table 1) that did not agree with PCR marker-based haplotypes. Haplotype data available from marker genotyping of the RIs is often used to correlate behavior with genotype, but in this particular case, the potential impact of the *Taar1* polymorphism would be missed using the marker data.

The availability of a non-functional native receptor provides a genetic tool for use in behavioral and biochemical studies, and for comparison to studies using KO mice that do not express TAAR1. As opposed to an engineered KO, it is possible that a receptor incapable of responding to agonists can still participate in protein-protein interactions that modulate other signaling pathways, including effects on signal transduction related to the association of TAAR1 and dopamine D2 receptors [38, 39]. Effects of Taar1-related changes on these interaction partners could account for MA-related behavioral differences.

Our previous report [12] indicates that the P77T variant in the D2 strain does not respond to MA and Fig 2 provides the first data comparing the effects of the putative physiological agonists, β-PEA and tyramine, on the function of the B6 and D2 strain TAAR1 receptors. Experiments were conducted using trace amine concentrations from 10 nM to 100 μM and maximal responses required micromolar concentrations, suggesting that the receptor is only partially activated under physiological conditions [1]. Clearly, the P77T SNP, which is at the TM2/extracellular (luminal) interface, has a deleterious effect on receptor responsiveness to endogenous or synthetic agonists. Previous reports have not included this natural mutation in site-directed mutagenesis or receptor modeling studies [24, 25]. The importance of this mutation is supported by data from behavioral experiments using recently obtained B6 and D2 mice, or mice derived from recent B6 X D2 F2 crosses (i.e., MAHDR and MALDR mice), demonstrating that the mutation is associated with increased MA drinking, MA-induced conditioned place preference, and confers a lack of sensitivity to aversive effects of MA and MA-induced hypothermia [12, 13, 40–42].

The functional consequences of numerous non-synonymous SNPs in the human *TAAR1* have not previously been described. However, non-synonymous SNPs in a receptor can have functional consequences by changing cellular signal transduction. For example, the SNPs in the human μ-opioid receptor alter cAMP-stimulated CRE transcription [43], ERK phosphorylation [44], and also decrease receptor desensitization following morphine pre-treatment [45]. Based on data from behavioral experiments with D2, B6, MAHDR and MALDR mice [12], we hypothesized that SNPs in the *hTAAR1* should affect receptor-coupled signal transduction. At least one SNP resulted in a sub-functional, as opposed to a non-functional receptor. There are no known animal models for this functional variant, but some of the human variants could produce the high MA intake phenotype seen in mice. The non- and sub-functional human variants could also predispose subjects to other deleterious MA-related consequences, as well as disorders related to other addictive substances and (or) other organ systems and diseases. Although breeding for ethanol-related behaviors has not resulted in any QTLs in the chromosomal region that includes *Taar1*, *Taar1* KO mice prefer ethanol in a 2-bottle choice paradigm, and are more sensitive to the sedative effects of ethanol [46]. The regulation of TAAR1 receptor expression has also been described in T cells [37] and the effects of exposure of primary human astrocyte cultures to MA and HIV-1 increases TAAR1 mRNA expression [47]. The consequences of SNP-induced structural or functional changes in TAAR1 on HIV-1-mediated receptor expression are unknown, but could result in altered cellular response to the viral infection. In addition to human astrocytes, TAAR1 is expressed on mouse T and B cells, and stimulation of T cells by β-PEA regulates T cell function as measured by changes in cytokine expression signal transduction intermediates [48, 49]. Thus, human subjects with sub- or non-functional lymphocyte TAAR1 may have altered sensitivity to circulating trace amines, as well as an altered inflammatory and immune response.

A number of additional phenotypic effects associated with TAAR1 have been identified. *Taar1* KO mice are less sensitive to doses of apomorphine that induce stereotypy [50]. In dopamine transporter KO mice with reduced synaptic dopamine, amphetamine and β-phenethylamine inhibit hyperactivity, possibly via TAAR1 activation. Furthermore, the receptor may play a role in the effects of drugs used to treat Parkinson’s disease [51, 52]. A role for TAAR1 in symptoms of chronic migraine, which is accompanied by significantly elevated levels of tyramine, has also been described [53]. In the periphery, TAAR1 receptors have been identified in cardiac ventricular tissue, and TAAR1 function has been implicated in cardiac arrhythmias. Recently, D2 mice have been identified as a model of familial hypertrophic cardiomyopathy [54] and these were likely D2 mice that possess the non-functional *Taar1* mutation. In addition, TAAR1 receptors are expressed in β-cells of pancreatic islets [55], and have been implicated in diabetes, obesity, and in kidney, liver, gastrointestinal and pancreas disorders [56, 57].

As the role of TAAR1 in pathology is characterized, and receptor-specific drugs are developed, personalized therapies based on the variants expressed by individual patients could improve treatment development and treatment outcomes. The loss of function conferred by some of the variants in the human receptor is also important for clinical drug development: agonist treatment of subjects expressing non-functional variants should have little or no effect, and prescreening subjects for non-functional receptors could reduce development time and expense, and improve productivity [58].
